# Magnetization Reversal Modes in Short Nanotubes with Chiral Vortex Domain Walls

**DOI:** 10.3390/ma11010101

**Published:** 2018-01-10

**Authors:** Ai Ping Chen, Julian Gonzalez, Konstantin Guslienko

**Affiliations:** 1 Department Materials Physics, Faculty of Chemistry, University of the Basque Country, UPV/EHU, 20018 San Sebastián, Spain; julianmaria.gonzalez@ehu.eus (J.G.); kostyantyn.gusliyenko@ehu.eus (K.G.); 2 IKERBASQUE, The Basque Foundation for Science, 48013 Bilbao, Spain

**Keywords:** magnetization reversal, nanotubes, magnetization hysteresis, domain walls, micromagnetic modeling

## Abstract

Micromagnetic simulations of magnetization reversal were performed for magnetic nanotubes of a finite length, *L*, equal to 1 and 2 μm, 50 and 100 nm radii, *R*, and uniaxial anisotropy with “easy axis” parallel to the tube length. I.e., we considered relatively short nanotubes with the aspect ratio *L*/*R* in the range 10–40. The non-uniform curling magnetization states on both ends of the nanotubes can be treated as vortex domain walls (DW). The domain wall length, *L_c_*, depends on the tube geometric parameters and the anisotropy constant *K_u_*, and determines the magnetization reversal mode, as well as the switching field value. For nanotubes with relative small values of *L_c_* (*L_c_*/*L* < 0.2) the magnetization reversal process is characterized by flipping of the magnetization in the middle uniform state. Whereas, for relative large values of *L_c_*, in the reverse magnetic field, coupling of two vortex domain walls with opposite magnetization rotation directions results in the formation of a specific narrow Néel type DW in the middle of the nanotube. The nanotube magnetization suddenly aligns to the applied field at the switching field, collapsing the central DW.

## 1. Introduction

One of the central problems in the area of nanomagnetism is proper description of the magnetization reversal, the reversal modes and corresponding reversal (switching) fields [1]. Magnetization reversal strongly depends on the sample shape and size, as well as on the magnetic material parameters. Experimentally, the magnetization reversal and switching fields are detected via measurements of magnetic hysteresis loops. Very often, the magnetization reversal mode description can be reduced to the motion of magnetic solitons (domain walls) and one (two) domain wall approximation is sufficient for satisfactory description of the reversal. On the other hand, recently, spintronic devices based on domain wall motion such as race-track memory [2] and magnetic logic gates [3] have been proposed (see the review in Ref. [4]). 

The simplest magnetic systems to study the domain wall motion are thin magnetic stripes, or wires with rectangular cross sections. The magnetization reversal in the stripes can be reduced to motion of vortex or transverse domain walls [5,6], so called “head-to-head” and “tail-to-tail” walls. Other typical systems important for applications are magnetic wires with a circular cross section [1,4]. However, the magnetization reversal in such wires is more complicated due to the presence of vortex cores (Bloch lines) and singular Bloch points near the wire center, resulting in an increase in the switching fields. To avoid the vortex cores, designs using hollow circular wires or tubes have been suggested, see Ref. [4,7] for the recent review. 

Magnetic tubes with nanoscale radii and thickness (nanotubes) are promising candidates for the development of nanoscale devices, and have been intensively investigated using experimental and theoretical methods over the last decade [8,9,10,11,12,13,14,15,16,17,18,19,20,21,22,23,24,25,26,27,28,29,30,31,32,33]. Furthermore, with the rapid development of measuring tools, such as the optimally coupled nanometer-scale SQUID combined with torque magnetometry [9] and dynamic cantilever magnetometry [10], the inhomogeneous magnetization configurations and switching modes can nowadays be probed in an individual nanotube. The internal field distribution is inhomogeneous, mainly due to the long-range magnetostatic interaction, and this essentially influences the reversal modes excited in various shapes of confined nanostructures, such as flat magnetic particles of different shapes (dots), nanowires, nanotubes, nanorings, etc. [1].

The magnetization hysteresis loops recently observed and simulated in Ni nanotubes by Buchter et al. [9] exhibited the same oblique parallelogram shapes as those simulated in short nanotubes with an uniaxial anisotropy with an “easy axis” directed along the tube length [23]. The authors of Refs. [9,10] pointed out that the magnetization reversal was not initiated from both ends of nanotube, as predicted in Refs. [19,20] for long nanotubes. According to the observations in [9,10], the magnetization reversal process is presumably composed of reversible and irreversible contributions. The reversible part corresponds to a Néel-type domain wall nucleation between two tubular-like vortex domains with opposite circulation directions as the increasing magnetic field approaches the switching field value and the magnetization gradually decreases, forming a special magnetization configuration. The irreversible reversal takes place by collapse of the Néel-type wall in the middle of the nanotube at the switching field.

In this work, we simulate the magnetization reversal by describing the intermediate magnetization states of nanotubes as the distorted vortex domain walls on both tube ends with the localization length *L_c_*. The magnetization reversal process is presented as the displacement of these walls, followed by the formation and collapse of a Néel-type domain wall in the middle of the nanotube. Since the length *L_c_* depends on both material and geometric parameters of nanotube, the magnetization reversal reveals various modes.

## 2. Methods

**Figure 1:**
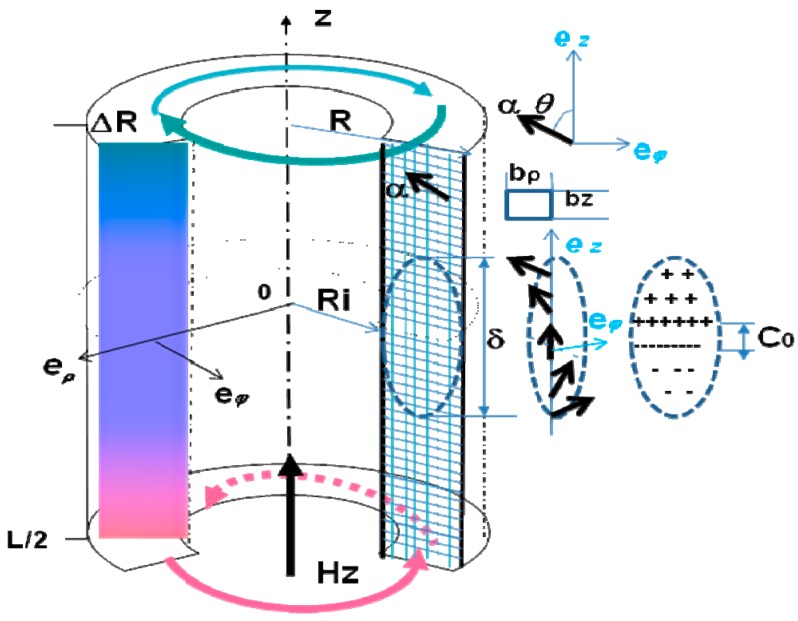
Circular magnetic nanotube with outer radius *R*, inner radius *R_i_*, length *L*, and the cylindrical coordinate (*ρ*, *φ*, *z*) system used. The simulations are performed in the nanotube cross-section of Δ*R* × *L*. The magnetization configuration of the domain wall located in the middle is illustrated in the right panel.

A cylindrical magnetic nanotube with outer radius *R*, inner radius *R_i_* and length *L* is set on the cylindrical coordinates system (*ρ*, *ϕ*, *z*) with the origin in the middle of tube and the *z* axis directed along the tube length, see Figure 1. The nanotube is assumed to have magnetic uniaxial anisotropy, with an easy axis parallel to the *z* axis. An external magnetic field (*H_z_*) is applied along the *z* axis opposite to the average magnetization of the central nanotube domain. 

One of the fascinating properties of the tubular geometry is its rotational symmetry; the fact that the magnetization configuration is identical in the tube cross-section with an arbitrary azimuth angle. This means that the magnetization configuration inside of a nanotube with a thickness ∆*R* = *R* − *R_i_* can be revealed by performing 2D numerical simulations in the rectangular area of the cross-section of ∆*R* × *L*. In the simulations, the material parameters adopted were: the exchange stiffness constant, *A* = 10^−11^ J/m, the saturation magnetization, *M_s_* = 10^6^ A/m, and the relatively small uniaxial anisotropy constant, *K_u_*, was equal to *K*_0_ = 5 × 10^3^ J/m^3^ or 2*K*_0_ = 10^4^ J/m^3^ for studying of the anisotropy effect. The choice of the material parameters determines the material intrinsic scales: the exchange length, $R_{0}={\left(4\pi A/\mu_{0}M_{s}^{2}\right)}^{1/2}$ = 10 nm and nominal domain wall width $\delta ={\left(A/K_{u}\right)}^{1/2}\approx$ 40 nm. 

The computations are implemented by the software Delphi 7 [34]. The tube cross-section ∆*R* × *L* is meshed with the cell size, as *b_ρ_* = *b_z_* = 2.5 nm, which is smaller than the exchange length *R*_0_. The magnetization at each cell is described by the unit vector ***α*** = ***M***/*M_s_*. Its distribution in the nanotube wall section ∆*R* × *L* can be represented by the 2D diagrams of vectors and colors, as shown in Figure 1, as well as by the components of the magnetization unit vector, $\alpha_{\varphi }=\sin\theta \left(z\right)$ and, $\alpha_{z}=\cos\theta \left(z\right)$, where *θ* is the angle between the magnetization direction and the tube easy axis *z*. 

According to the theoretical studies [18,20], the exchange energy is associated with the nanotube curvature factor, ∆*R*/*R* or *β* = *R_i_*/*R*. In order to elucidate the effect of exchange interaction on the magnetization configurations, the simulations are carried out in nanotubes of 50 nm and 100 nm radii with varying tube thickness ∆*R*. 

## 3. Results of Simulations

### 3.1. Zero External Fields

**Figure 2:**
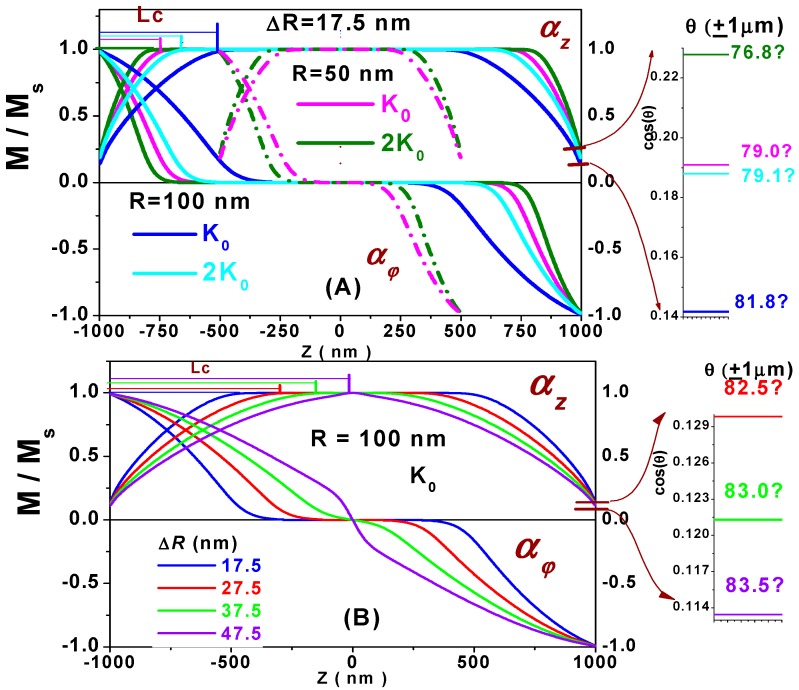
Simulated magnetization configurations of nanotubes for *H_z_* = 0. (**A**) the fixed tube thickness Δ*R* = 17.5 nm. The nanotube radius *R* = 50 nm and tube length 1 μm (dashed lines) and 2 μm (solid lines): magnetic anisotropy constant *K_u_* values *K*_0_ = 5 × 10^3^ J/m^3^ (pink lines) and 2*K*_0_ (green lines). The nanotube radius *R* = 100 nm and length 2 μm: *K*_0_ (blue lines), 2*K*_0_ (cyan lines); (**B**) nanotube with a length of 2 μm and a radius of 100 nm, and the anisotropy constant *K*_0_ with tube thicknesses Δ*R* = 17.5, 27.5, 37.5 and 47.5 nm, represented by the blue, red, green and violet curves, respectively.

The simulated magnetization configurations of the thin nanotubes, ∆*R* = 17.5 nm, with 50 and 100 nm radii, and the nanotubes of *R* = 100 nm with varying tube thickness are presented respectively in the upper and lower panels of Figure 2. 

The magnetization configurations characterized by the non-uniform states with decreasing of the *α_z_* value towards both ends of the nanotube (twisted “bamboo” states [11]) are referred to as partial Néel-type domain walls (DWs) [18] or vortex end-domains [19,20]. For the relatively thin and short nanotubes, axially symmetric DW is formed at the middle to separate two vortex-like domains with the magnetization configurations having opposite chiralities. Within the DW width, the magnetizations rotate from one circular sense to another, resulting in a special DW configuration composed of a core and two tube end-domains. The core with length *C*_0_ is characterized by magnetizations aligning to the positive *z* axis and the magnetic charges with the maximum density concentrated on its surfaces, while magnetic charges of a definite sign are distributed respectively within two end-domains as a consequence of the magnetization rotation. The width of these axially symmetric Néel-type DWs is strongly dependent on the nanotube sizes [31]. The formation of the chiral DWs with magnetization circulation in opposite directions minimizes the total energy of the nanotubes in the following way: to minimize the magnetostatic energy, the magnetic moments located on the nanotube end faces z=±L/2 experience a torque exerted by the demagnetizing field to rotate an angle, $\theta_{end}=\pm \theta \left(\mp L/2\right)$, tilting away from the tube easy axis, which is held by the exchange and anisotropy interaction to avoid increases of these two energy values. The balance between these energy terms is reflected in the value of the angle $\theta_{end}$ < 90°, as illustrated by the magnified sketch in the right panel of Figure 2. 

Due to the dependence of these energy terms on the nanotube material and geometric parameters, the deviating angle $\theta_{end}$ exhibits a dependence on the anisotropy constant *K_u_* as well as on the nanotube sizes. For example, for nanotubes of the same material and length, the manetostatic energy is considered to be proportional to the area of the nanotube end face, which is proportional to the tube radius *R* and thickness ∆*R*, so that by increasing the radius from 50 to 100 nm, the angle $\theta_{end}$ increases from 79° to 82° for the anisotropy constant value *K*_0_, and from 77° to 79° for 2*K*_0_, as shown in the upper right panel in Figure 2. Whereas for nanotubes of 100 nm radius, as the thickness increases from 17.5 to 27.5, 37.5 and 47.5 nm, the angle $\theta_{end}$ increases from 82° to 82.5°, 83° and 83.5°, respectively, as displayed in the bottom right panel of Figure 2. On the other hand, the anisotropy field prevents the magnetization rotation. This is demonstrated by the fact that the angle $\theta_{end}$ decreases as the anisotropy constant value increases from *K*_0_ to 2*K*_0_ for the same nanotube sizes.

Another factor preventing magnetization rotation is the exchange interaction, which is considered to be proportional to the tube curvature factor, ∆*R*/*R*. As can be seen in the upper panel of Figure 2, for the same nanotube thickness ∆*R* = 17.5 nm, the angle $\theta_{end}$ is smaller for *R* = 50 nm than that for *R* = 100 nm. For the latter, the superposition of the larger stray field and the smaller exchange effect makes the difference between these two values of $\theta_{end}$ quite small. However, for the same radius of nanotubes, the magnetostatic and the exchange energies are both proportional to ∆*R*. The fact that the value of the angle $\theta_{end}$ increases with increasing ∆*R* is exhibited in the lower panel of Figure 2 for nanotubes of *R* = 100 nm. This means that the magnetostatic energy, as a long-range interaction, has an advantage over the short-range exchange interaction in the energy competition. When the nanotube thickness ∆*R* increases to 47.5 nm, non-uniform states extend to the middle of the tube. The purple curves show that the magnetization rotates from +12° to −12° in the central region around 150 nm. It means the nucleation of the domain wall in the center of the nanotube.

The domain wall length *L_c_*, designated as the distance through which the magnetization progressively rotates from the angle $\theta_{end}$ to align with the easy axis, also results from the energy competition and balance in the lowest energy state. To minimize the magnetostatic and exchange energies, the magnetization rotation is realized by making the angles between adjacent magnetic moments as small as possible, which should be restrained by the anisotropy interaction, avoiding extension of the rotational region and increasing the anisotropy energy. As shown in Figure 2, the length *L_c_* of DW is always much larger than δ, is proportional to the tilting angle $\theta_{end}$ of the magnetization at the edge of the nanotube, and demonstrates dependencies similar to those of the angle $\theta_{end}$ on the values of the material parameter *K_u_*, and the geometric parameters of nanotube, including the radius *R*, length *L* and the thickness ∆*R*, as discussed in detail above.

**Figure 3:**
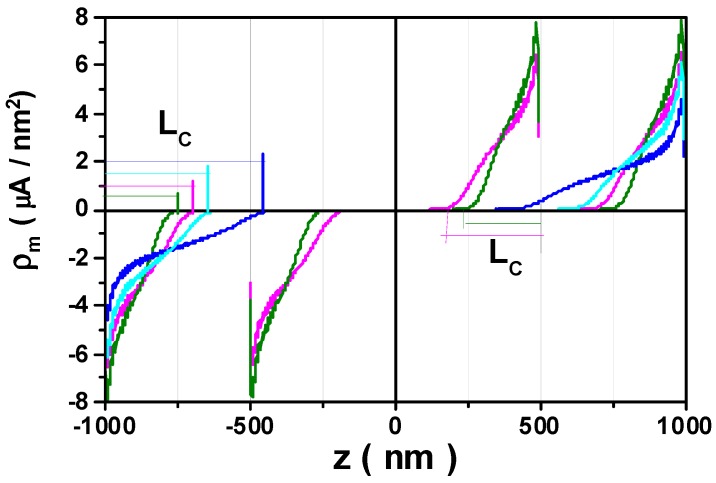
Simulated magnetic charge distribution in nanotubes with the lengths of 1 and 2 μm: for the nanotube radius *R* = 50 nm, the anisotropy constant values, *K*_0_ (pink lines) and 2*K*_0_ (green lines); for the nanotube radius *R* = 100 nm, the anisotropy constant values, *K*_0_ (blue lines) and 2*K*_0_ (cyan lines). The *L_c_* symbols mark the sizes of the vortex-like end domains.

On the other hand, the Néel-type domain wall with the magnetization rotations from the angle $\theta_{end}$ to alignment with the easy axis gives rise to the magnetic volume charges within the DW length *L_c_*. This can be calculated following the formula $\rho_{m}=-\nabla \cdot M=-M_{s}\left(\alpha_{\rho }/\rho +d\alpha_{z}/dz\right)$. We found that the *ρ*-magnetization component is negligibly small for all simulated nanotube sizes. Nevertheless, this component is important for DW dynamics, resulting in a chirality-dependent DW velocity in long nanotubes [24,25,30]. Figure 3 demonstrates the calculated values of $\rho_{m}$ (μA/nm^2^), distributed in thin nanotubes with a 50 nm radius and lengths of 1 μm and 2 μm, as well as in nanotubes with a 100 nm radius and 2 μm length. Obviously, the negative and the positive magnetic charges appearing on the length *L_c_* correspond to the magnetization rotation from $\theta_{end}$ to 0° near the tube bottom face, and from 0° to −$\theta_{end}$ near the top face of the tube, respectively.

### 3.2. Magnetization Reversal Process

**Figure 4:**
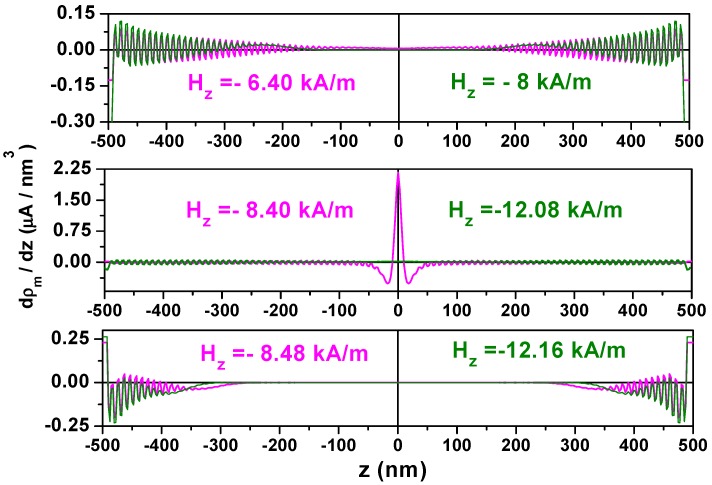
Magnetic charge distribution applying the reversed magnetic field *H_z_* for nanotubes with thickness ∆*R* = 17.5 nm, radius *R* = 50 nm and 1 μm length using the magnetic anisotropy constants *K*_0_ and 2*K*_0_ plotted with the magenta and olive colours, respectively.

In the magnetic fields *H_z_* applied along the tube axis, the magnetic moments experience a torque, **Г** = **M** × **H** = $M_{s}H_{z}\alpha_{\varphi }\left(z\right)\hat{\rho }$, to set magnetization precession around the field direction, leading to domain growth from both tube ends towards to the tube middle. The torque is not only dependent on the strength of the applied magnetic field, but also on the magnetization component $\alpha_{\varphi }\left(z\right)$, which leads the amplitude of the torque to have the same dependence on the coordinate *z* as $\alpha_{\varphi }\left(z\right)$. End-domain growth is mainly influenced by the anisotropy field, 2*K_u_*/*M_s_*, determined by the anisotropy constant value. For nanotubes of 50 nm radius and 1 μm length, Figure 4 compares the magnetic charge distribution for different bias fields in the nanotubes with the different anisotropy constant values, *K*_0_ and 2*K*_0_, plotted with the magenta and olive color, respectively.

For nanotubes with larger values of anisotropy (2*K*_0_), the magnetic charge distribution (olive color) reveals a shorter relative localization length. They are only localized in about one quarter of the nanotube length at the applied magnetic fields of −12.08 kA/m. By increasing the magnetic field strength by 0.08 kA/m, the magnetization reversal occurs, resulting in most of the magnetic moments being aligned with the reverse field direction. However, there is still a small amount of charge localized at both ends of the tube, due to the effect of the edge demagnetizing field. For the nanotube with the smaller value *K*_0_, the magnetic charge localization length is relative larger. At the magnetic field strength of −6.4 kA/m, the charges (magenta color) already exist around the tube center. In the field of −8.40 kA/m, the central domain contraction results in the sharp maximum of the charge density at the middle of nanotube. This is manifested by increasing the charge density by approximately 20 times. Only a small increase in the magnetic field strength of 0.08 kA/m is needed to reach the switching field value, −8.48 kA/m. The magnetization suddenly reverses and aligns in the central tube area to be parallel with the reversed field direction. We note that the magnetization charge density $d\rho_{m}/dz=-M_{s}\left(d^{2}\alpha_{z}/dz^{2}\right)$ is proportional to the *z*-component of the exchange field and, therefore, reflects the exchange energy density. 

**Figure 5:**
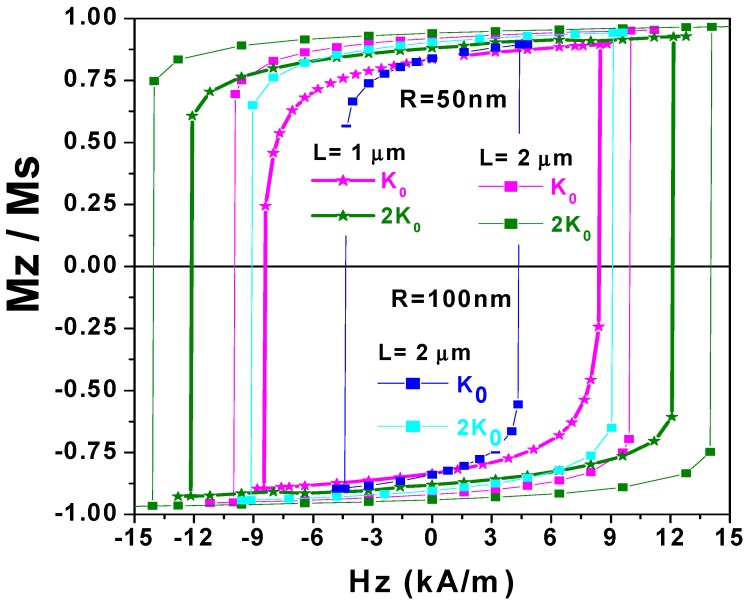
Simulated magnetization hysteresis loops of thin nanotubes with Δ*R* = 17.5 nm. Nanotube length *L* = 1 μm, radius *R* = 50 nm: magnetic anisotropy *K_u_* values *K*_0_ = 5 × 10^3^ J/m^3^ (pink stars) and 2*K*_0_ (green stars). Nanotube length *L* = 2 μm: *R* = 50 nm, *K*_0_ (pink rectangles), 2*K*_0_ (green rectangles); *R* = 100 nm, *K*_0_ (blue rectangles), 2*K*_0_ (cyan rectangles).

The magnetization reversal mode is also reflected in the hysteresis loops, as presented in Figure 5. The growth of the vortex end-domains leads to a reduction in the average value of the axial magnetization component, *α_z_*. For the magenta curve for the nanotube with *L* = 1 μm at the field value of −8.40 kA/m, the nucleation of the central DW is manifested by decreasing the values of *M_z_*/*M_s_* to its lowest value. A similar magnetization reversal mode is observed in the 2 μm-long nanotube with 100 nm radius and the smaller anisotropy constant value of *K*_0_ plotted in Figure 5 (blue color). Another reversal mode displayed by the olive star hysteresis loop is the same as that plotted with small olive squares, and magenta and cyan colors. It is also shown in Figure 5 that the switching field value, *H_s_*, can be controlled not only by the anisotropy value *K_u_*, but also by changing the nanotube geometry. 

**Figure 6:**
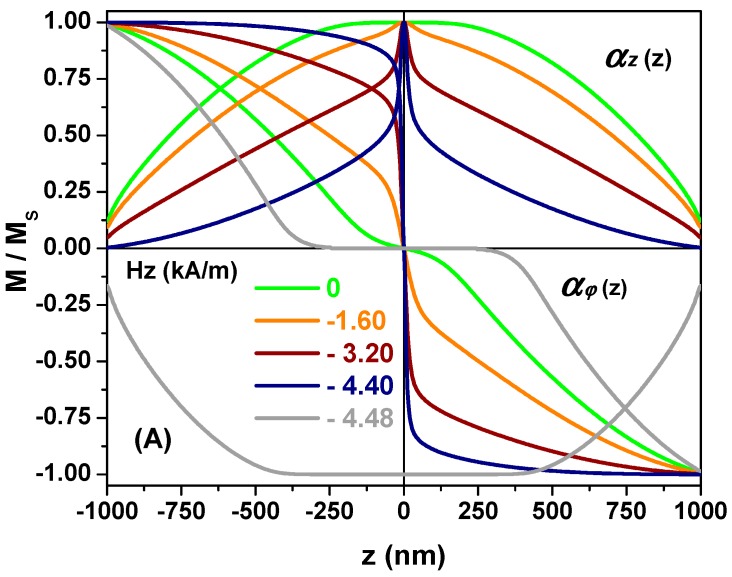

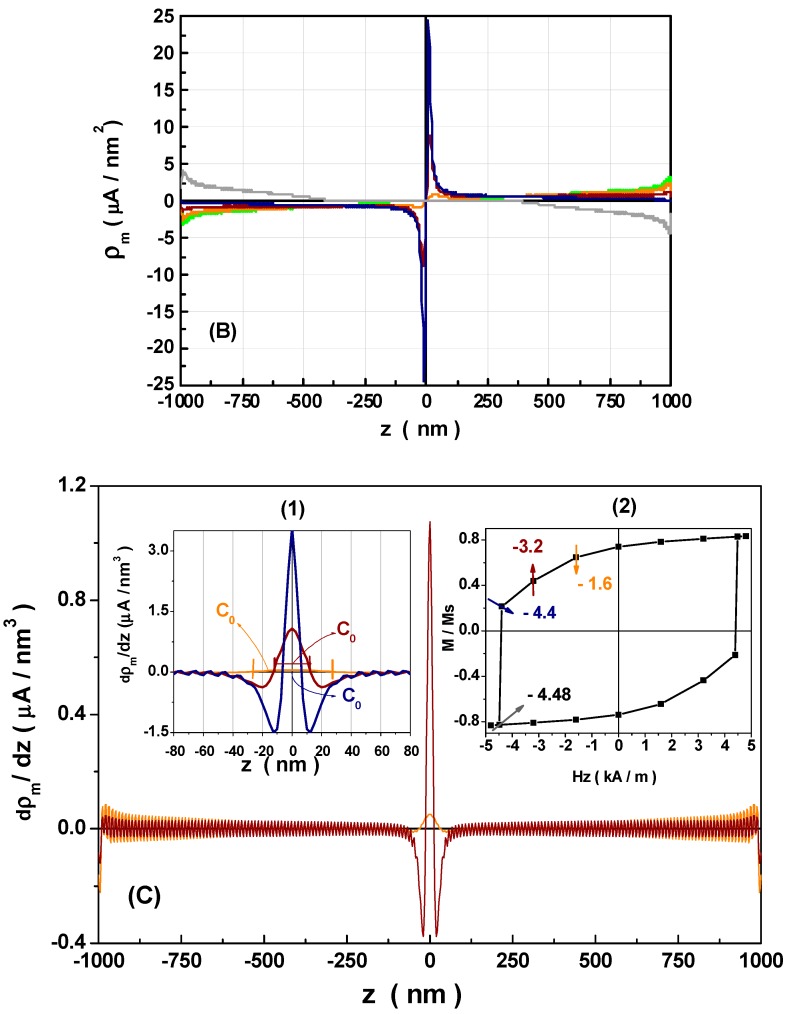
The magnetization component profiles and magnetic charge distributions within a nanotube with the applied magnetic fields of 0, −1.6, −3.2, −4.40 and −4.48 kA/m represented by the green, orange, brown, navy, blue and gray color curves, respectively: (**A**) the magnetization components, $\alpha_{\varphi }\left(z\right)$, $\alpha_{z}\left(z\right)$, profiles; (**B**) magnetic charge distributions; (**C**) derivative of the magnetic charges. Inset (1): *C*_0_ marks the core width of the central domain wall in the middle of nanotube. Inset (2): simulated magnetization hysteresis loop showing the average magnetization for the selected values of the magnetic fields used in panels (**A**,**B**). The nanotube geometrical parameters are: radius *R* = 100 nm, thickness 37.5 nm and length *L* = 2 μm.

The magnetization process exemplified by Figure 4 and Figure 5 starts from both ends of the nanotube by the growth of the vortex domains. However, if the uniform state, with magnetization parallel to the easy axis, occupies only a small middle part of the nanotube, the magnetization reversal mode exhibits another feature. See, for example, the magnetization reversal stages for the nanotube with length 2 μm, radius 100 nm and thickness 37.5 nm, as presented in Figure 6.

At the zero field, the green magnetization distribution curves, $\alpha_{\varphi }\left(z\right)$ and $\alpha_{z}\left(z\right)$, demonstrate the uniform state appearing in the middle of the nanotube within the range of ±125 nm (Figure 6A). By applying a small reversed field of −1.6 kA/m as shown by the orange curves in the middle, the uniform magnetization state is replaced by the rotation of the magnetization from θ≈ 22° ($\alpha_{\varphi }=\sin\theta$ = 0.375) to θ≈ −22°. This leads to the appearance of magnetic charges within the domain wall located at the center of the nanotube. The structure of the central DW, based on magnetic charges distribution curves, $\rho_{m}\left(z\right)$, can be assumed to consist of a core with the maximum of the negative and the positive magnetic charges concentrated on its surfaces, and two regions distributed with different signs of magnetic charges, as sketched in the right panel of Figure 1.

When the reverse magnetic field is increased to −3.2 kA/m, the end-domain growth towards to the middle of the nanotube undergone by the two domains magnetized in the opposite circular directions compresses the central domain wall and causes the magnetization rotation from 46° to −46° at the middle region of ±125 nm, giving rise to the giant value ±8.94 μA/nm^2^ of the magnetic charges on the core surfaces. The core length *C*_0_, which can be measured as the distance between two points, where $d\rho_{m}\left(z\right)/dz=0$, is 40 nm, as shown in Figure 6C by the brown curve in the magnified sketch of the magnetic charge density $d\rho_{m}\left(z\right)/dz$ on the scale of ±50 nm. 

As the reversed field increases to −4.4 kA/m, the navy blue curves demonstrate that the magnetization rotation from 63° to −63° is suppressed at the central narrow region of ±100 nm, providing information about the dramatic increases in the magnetic charges (and the exchange energy density) within the DW, and shrinking of the core length to 25 nm. 

By further increasing the reverse field to 4.48 kA/m, the magnetization reversal takes place by collapsing the central narrow DW. The gray curves demonstrate the central nanotube domain, where the magnetizations align to the reverse field direction, which becomes longer and has a length about of 750 nm. In the reversed state, the nanotube edge effect can still be observed by rotating the magnetizations progressively from 0° to 80.5° towards the two ends of the nanotube. In the field above the reversal field, the magnetic charge sign at both ends of the nanotube is completely opposite to the zero-field state plotted with the green curve. The uniformly magnetized central domain is longer than the one in the zero fields. Therefore, the magnitude of the average axial magnetization component, *M_z_*/*M_s_*, is equal to 0.827, as indicated by the hysteresis curve, which is larger than 0.739 in the initial magnetization state at *H_z_* = 0. 

Therefore, the magnetization reversal process of the nanotube with such a magnetization structure can be characterized by the central DW forming, the DW shrinking, and then collapsing at the switching fields. The magnetization reversible reversal process still starts from both tube ends due to growth of the vortex domain walls decreasing the average axial magnetization component. The irreversible magnetization reversal is manifested itself by aligning of the magnetic moments in the central domain parallel to the reversed field direction and disappearing of the central domain wall between oppositely circulating magnetic domains. The reversal mode is specific for the nanotube topology and does not exist in “unrolled” nanotubes (magnetic stripes), where the magnetization reversal is mediated by magnetic vortices and antivortices nucleated at the stripe lateral edges. Such mode of the magnetization reversal of short nanotubes with the opposite chiralities of the vortex end-domains was experimentally confirmed for anisotropic Ni [9], exchange-biased permalloy [10] and CoFeB nanotubes [32].

## 4. Summary

Based on the simulation results, we can draw the following conclusions: (1) Neél-type vortex domain walls of opposite chirality are formed in finite magnetic nanotubes to minimize the total magnetic energy following the principle of magnetic charge avoidance; (2) the domain wall length *L_c_* is determined by the angle that the magnetization deviates from the easy axis at both ends of the nanotube; (3) *L_c_* plays an essential role in the magnetic properties of nanotubes, and it can be tailored by adjusting the tube geometry and the anisotropy constant *K_u_*; (4) for a finite length of nanotubes the reversible part of the magnetization reversal is initiated at both ends of the nanotubes, with the vortex domain wall growth decreasing the axial component of magnetization. For nanotubes with a relatively shorter length of the vortex wall located at both tube ends (*L_c_*), the irreversible magnetization reversal occurs by overcoming the energy barrier contributed mainly by the anisotropy energy. Whereas, for nanotubes with relatively large value of *L_c_*, the irreversible part of the magnetization reversal is realized by collapsing the central Néel domain wall.
